# Refining Frenectomy by Miller’s Technique: A Case-Based Overview

**DOI:** 10.7759/cureus.87967

**Published:** 2025-07-15

**Authors:** Neha Sahu, Supriya Mishra, Vineeta Gupta, Sonal L Kumbare, Alka Snehil

**Affiliations:** 1 Department of Periodontology, Government Dental College, Raipur, Raipur, IND; 2 Department of Periodontics, Government Dental College, Raipur, Raipur, IND

**Keywords:** aberrant maxillary labial frenum, frenectomy, laterally displaced flap, miller’s technique, primary intention healing

## Abstract

An aberrant labial frenum can lead to midline diastema and gingival recession, affecting both the aesthetic appearance and functional integrity of the oral cavity. Several treatment approaches are available for managing such frenum abnormalities. The traditional frenectomy method is a more invasive procedure that often results in visible scarring and the loss of the interdental papilla, compromising aesthetics. To address these limitations, Miller proposed a modified technique that combines frenectomy with a laterally displaced flap. This approach offers the advantage of healing by primary intention, significantly reducing the risk of scarring. This article presents a case report utilizing Miller’s technique for frenectomy. This case report presents a detailed account of Miller’s technique for a frenectomy procedure, including a follow-up conducted two weeks post-operation.

## Introduction

The frenum is an anatomical entity, etymologically derived from the Latin term fraenum, consisting of a membranous fold composed of mucous membrane, connective tissue, and occasionally, muscle fibers. Multiple frena are present within the oral cavity, with the maxillary labial frenum, mandibular labial frenum, and lingual frenum being the most frequently observed [1].

The frenum primarily functions to provide structural stability to the upper and lower lips as well as the tongue. However, when abnormally positioned, particularly when attached too closely to the gingival margin, they may contribute to gingival recession. This pathological condition can arise either from muscular tension that opens the gingival sulcus or from mechanical trauma due to improper toothbrushing technique [2].

Embryologically, the maxillary labial frenum develops from a post-eruptive remnant of the ectolabial bands, which connect the tubercle of the upper lip to the palatine papilla. In instances where the maxillary central incisors erupt with significant spacing, bone deposition beneath the frenum may be absent, leading to a V-shaped osseous cleft and an atypical frenum insertion. Similarly, a mandibular frenum is classified as aberrant when it is associated with a shallow vestibule and insufficient width of attached gingiva [3].

Four types of frena were distinguished by Placek et al. (1974) according to the anatomical position of their fiber implantation. Both the papillary and papilla-penetrating types are regarded as pathological because they are frequently linked to a number of clinical problems, including gingival recession, loss of the interdental papilla midline diastema, difficulty maintaining oral hygiene, and poor denture fit or retention. The mucosal type has fibers that attach at the mucogingival junction, while the gingival type has fibers that insert within the attached gingiva papilla and extend into the interdental papilla [4]. This can have a detrimental effect on one's mental and dental health, including gingival recession, interdental papilla loss, midline diastema, tooth malalignment, impaired oral hygiene practices, and compromised denture fit or retention, all of which may contribute to psychological distress [5].

According to Miller, a frenum should be deemed pathogenic if it is abnormally broad, lacks an adequate zone of attached gingiva at the midline, or causes displacement of the interdental papilla upon activation [6,7]. The clinical diagnosis of an aberrant frenum can be established through visual and functional examination. This involves retracting the upper lip outward and downward (or the lower lip outward and upward) and observing for movement of the gingival margin or ischemic blanching. A positive response to this maneuver, known as the blanching test or tension test, indicates an aberrant frenum [8].

According to Olivi et al. (2010), clinical indications for frenum excision arise in various scenarios, including the presence of an abnormal frenum that contributes to gingival inflammation due to impaired oral hygiene or a pathological frenum linked to inflammatory gingival recession. Furthermore, a noticeable maxillary labial frenum that remains after permanent canines have fully erupted and causes a midline diastema may need to be removed. Likewise, abnormal maxillary frena categorized as Class III or IV that cause midline diastema throughout the mixed dentition phase are also legitimate reasons to have surgery [9].

Management of these aberrant frena involves either frenectomy or frenulotomy procedures. Frenectomy refers to the complete excision of the frenum along with its bony attachment, whereas frenotomy entails repositioning the frenal attachment to a more favorable anatomical location [10].

One of the primary limitations of the traditional frenectomy technique is the formation of fibrotic scar tissue, which may lead to periodontal complications and compromise aesthetic outcomes.

To overcome these challenges, Miller proposed a modified approach that integrates frenectomy with a laterally positioned pedicle graft. This technique ensures midline closure using adjacent gingival tissue, thereby promoting healing by primary intention, which minimizes scar formation and enhances aesthetic results. Importantly, the interdental papilla remains intact since the trans-septal fibers are not disrupted, yielding superior functional and cosmetic outcomes [11].

This article presents a clinical case report highlighting the application of Miller’s technique in performing a frenectomy.

## Case presentation

**Figure 1:**
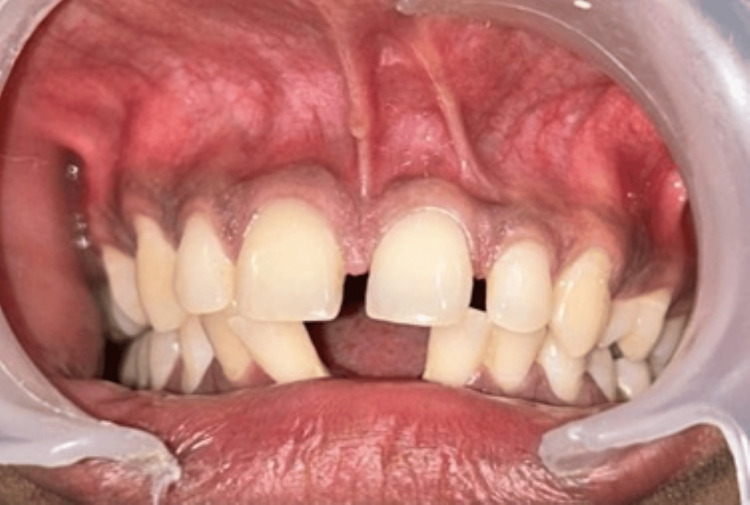
Pre-treatment intraoral photographs: frontal view

A clinical examination of a 27-year-old male patient who had come to the department of periodontology for evaluation showed an aberrant labial frenum, which was characterized by an abnormally broad attachment and an inadequate width of attached gingiva along the midline, in accordance with Miller's classification of an abnormal frenum (Figure 1). A positive tension test further supported the diagnosis in light of these findings. In order to address both practical and aesthetic concerns, a frenectomy utilizing the Miller technique was planned. Prior to the intervention, the patient was fully told about the surgical operation, including any possible risks and benefits, and written informed consent was acquired.

**Figure 2:**
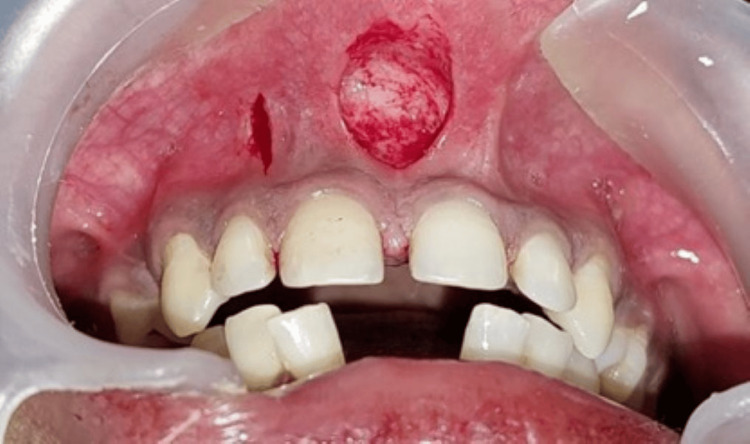
A vertical incision was made on the frenum, and a second parallel incision was made on the mesial side of the lateral incisor 2-3 mm apical to the marginal gingiva up to the vestibular depth

Under local anesthesia administered via infiltration with 2% lignocaine containing 1:80,000 adrenaline, a vertical incision was made and extended apically to the depth of the vestibule to completely detach the frenum from the alveolar mucosa. Any residual frenum tissue present along the midline and on the inner surface of the lip was excised (Figure 2). The procedure was carried out using standard periodontal instruments, including a No. 15 surgical blade, hemostat, sterile gauze, 5-0 black silk sutures, needle holder, suture scissors, and periodontal dressing.

**Figure 3:**
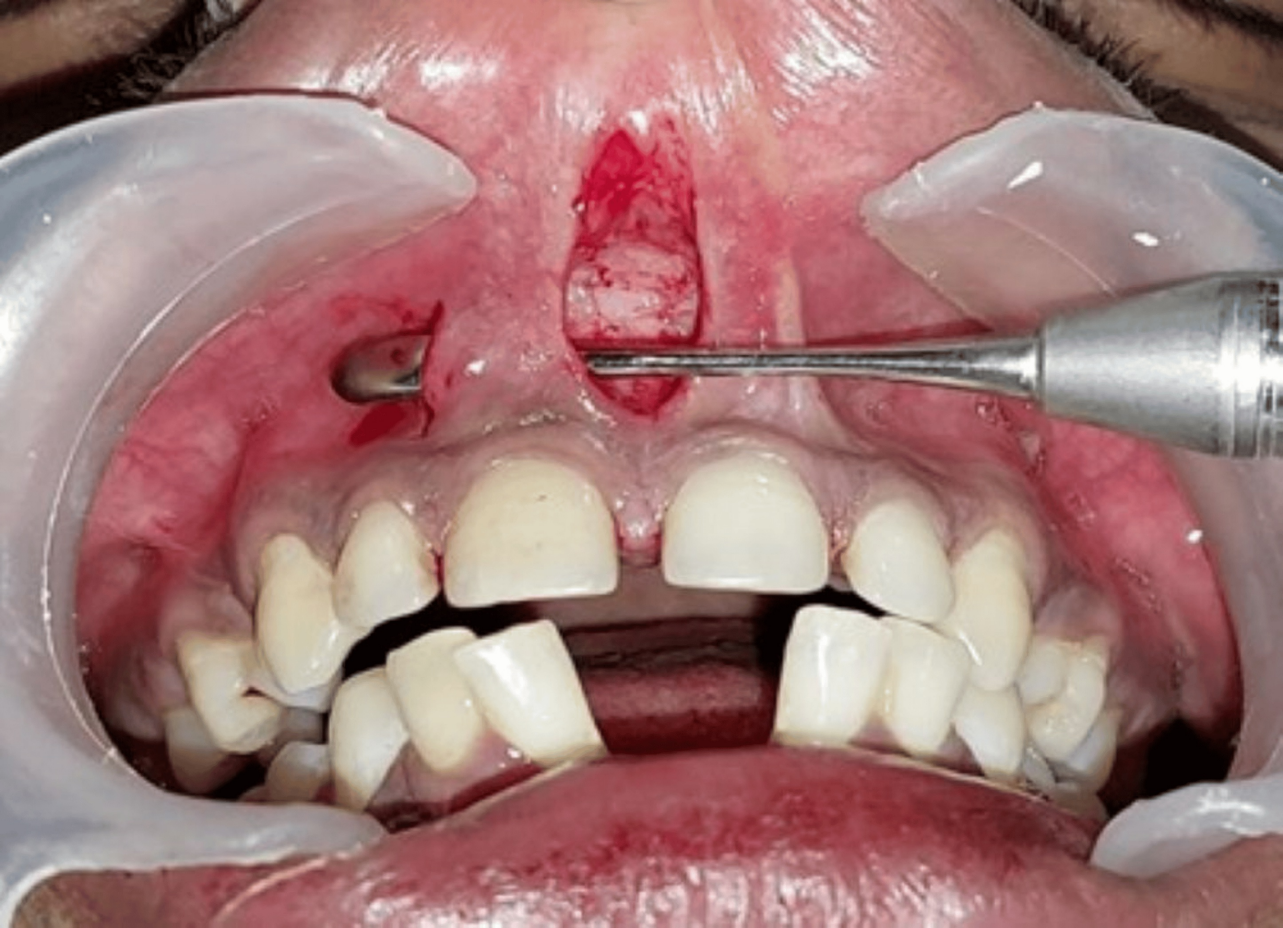
The gingiva and alveolar mucosa in between these two incisions were undermined by partial dissection to raise the flap

A parallel incision was placed approximately 2-3 mm apical to the marginal gingiva on the mesial aspect of the lateral incisor, extending toward the vestibular depth. The gingiva and alveolar mucosa situated between the horizontal and vertical incisions were partially undermined to elevate a flap (Figure 3).

**Figure 4:**
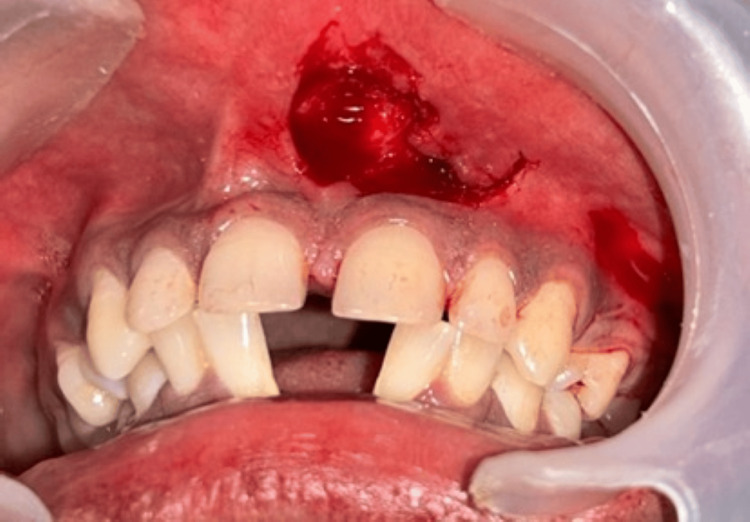
To separate the frenulum from the interdental papilla, a horizontal incision was made

**Figure 5:**
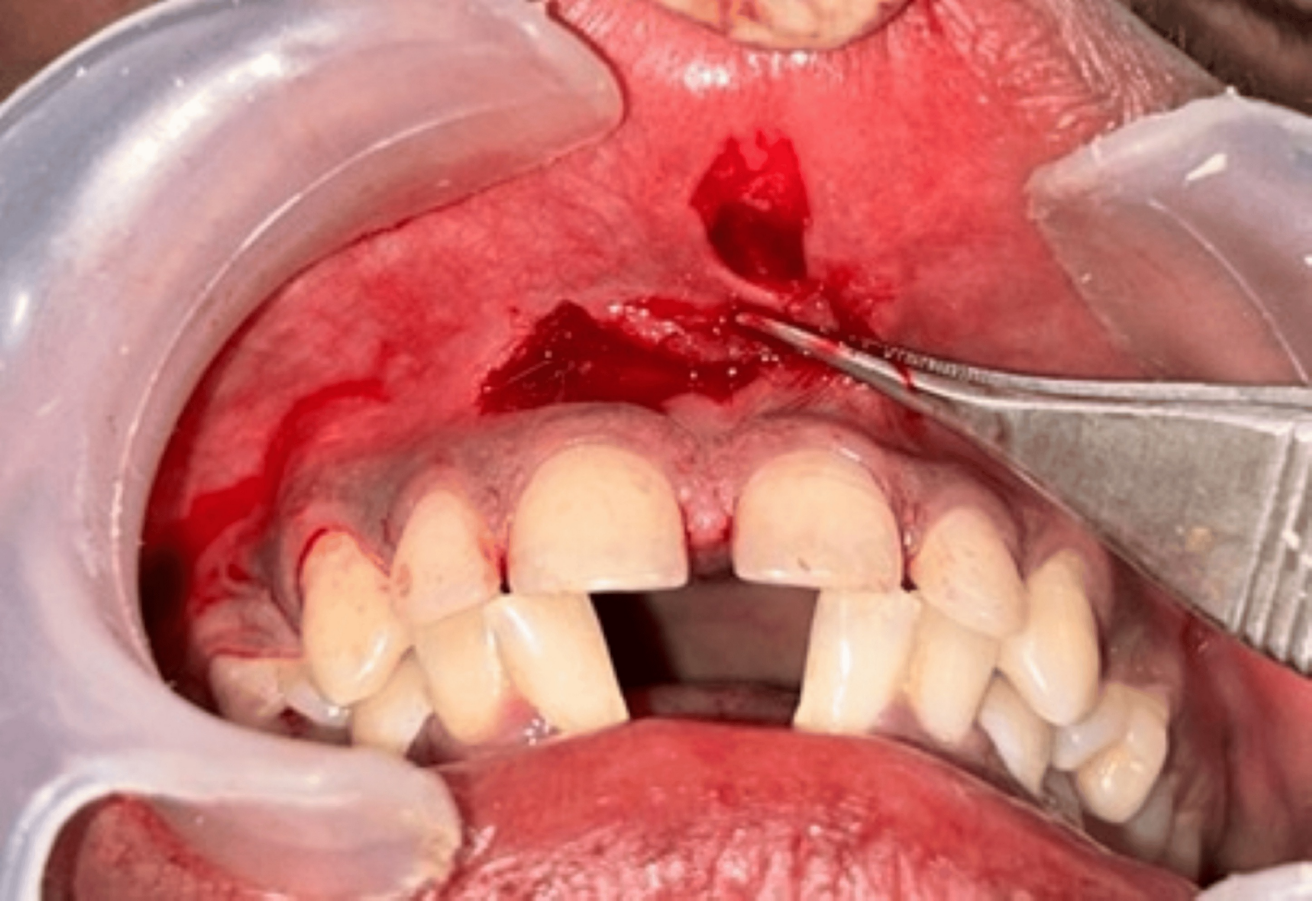
The flap was raised and mobilized mesially

A horizontal incision was made to detach the frenulum from the interdental papilla, extending apically to the depth of the vestibule to ensure complete separation of the frenum from the alveolar mucosa, approximately 1-2 mm apical to the gingival sulcus, connecting the coronal aspects of the two vertical incisions (Figure 4). The flap was raised and mobilized mesially (Figure 5). Any residual frenal fibers present along the midline and on the undersurface of the upper lip were carefully excised.

**Figure 6:**
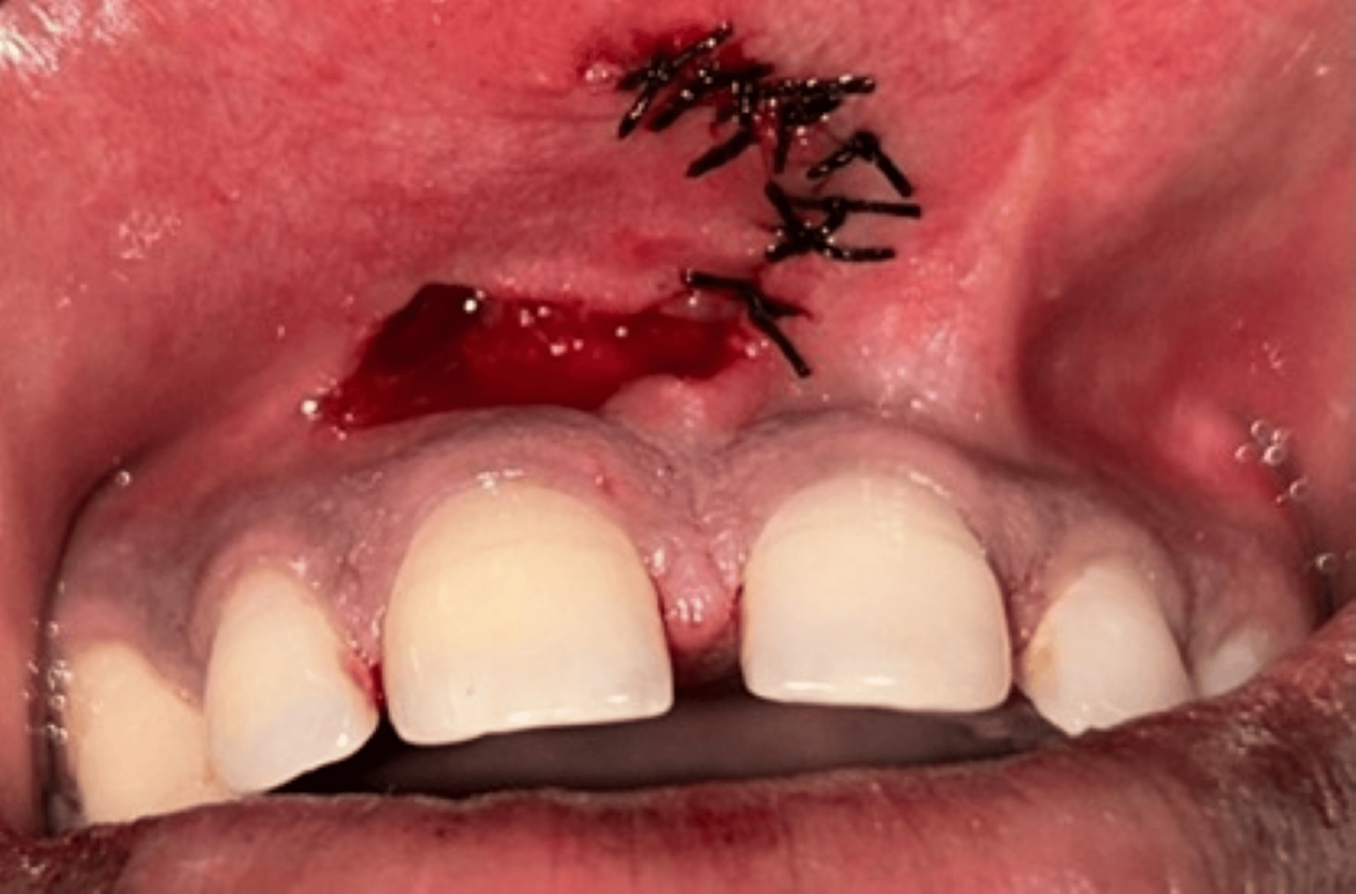
Sutured to obtain primary closure across the midline

**Figure 7:**
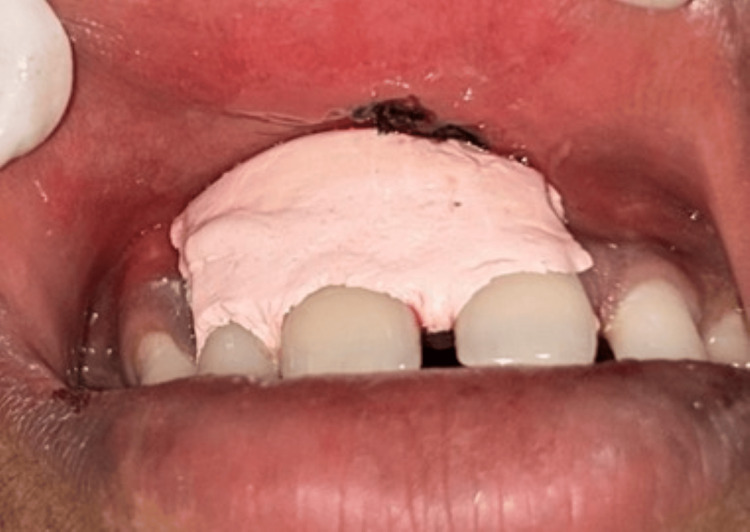
Periodontal coe-pack placed

**Figure 8:**
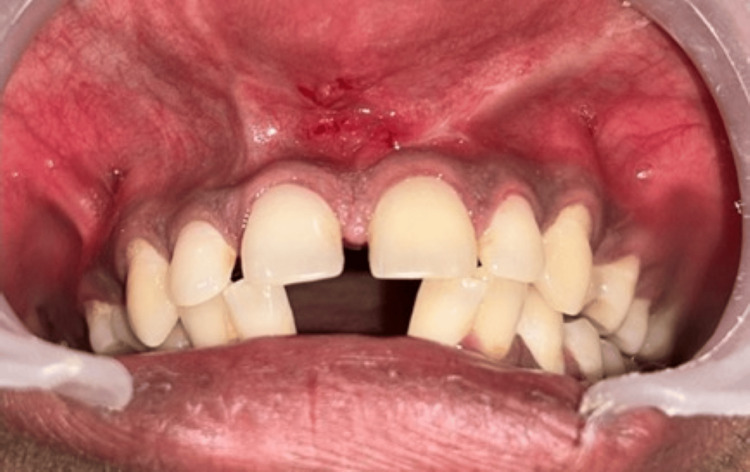
Two-week postoperative follow-up

The resulting flap was mobilized medially and sutured across the midline to achieve primary closure. 6-0 silk sutures were used, and simple interrupted sutures were given (Figure 6). A periodontal Coe-Pak dressing was applied at the surgical site to protect the wound and promote healing (Figure 7). Standard postoperative instructions were provided, and the patient was scheduled for a two-week follow-up for suture removal (Figure 8).

Postoperative care and follow-up

The patient was asked not to consume any hot and spicy food. The patient was prescribed an analgesic for three days and was recalled after seven days for suture removal.

## Discussion

A desire for more conservative and accurate methods that produce better functional and aesthetic results is increasing in the field of periodontal plastic surgery today. The treatment of abnormal frenal attachments has changed throughout time, moving from Archer's traditional frenectomy method to more sophisticated contemporary methods like those Edwards pioneered. The development of frenectomy techniques to reduce postoperative scarring and promote wound healing has been greatly aided by innovations such as soft tissue grafting and laser-assisted surgery [12]. Traditional frenectomy methods may result in severe intraoperative bleeding and increased patient morbidity due to postoperative problems such as substantial tissue damage from wide incisions [13].

The surgical procedure used by Miller, namely frenectomy with a lateral pedicle graft, is described in the case report paper by Bhosale N et al. The development of a thick zone of connected gingiva, color compatibility with surrounding tissue, healing by primary intention, and scar-free results are recognized as the principal benefits of the above strategy [14]. Important anatomical characteristics used to assess the frenum in this study are vestibular depth, attached gingiva width, interdental papilla health, and the existence or absence of midline diastema. In addition to being essential for maintaining a good cosmetic look, a sufficient zone of connected gingiva also helps to prevent gingival recession, which frequently requires surgical correction when it is compromised. 

When a frenum is too large, has no gingiva connected in the midline, or moves the interdental papilla when stretched, it is deemed abnormal. In research by Miller, a frenectomy and a laterally positioned pedicle graft were performed on 27 patients with such frena who had undergone orthodontic closure of a midline gap. In every instance, the interdental papilla was preserved during the operation. Only three of the 27 patients experienced modest reopening of less than 1 mm, and 24 of them showed no return of the gap. Miller proposed that stability resulted from the formation of a broad, collagen-rich band of connected gingiva, which probably assisted in keeping the closed gap in place. He emphasized that tooth mobility can occur, and roughly six weeks before appliance removal is the best time for this surgery.

In the present case report, postoperative evaluation confirmed an esthetically favorable result devoid of scarring. Pain levels were assessed using the Visual Analogue Scale (VAS) during the procedure and at 24 and 72 hours postoperatively. Mild to moderate discomfort was noted initially but subsided within two days. At the 48-hour postoperative review, the surgical site was asymptomatic, indicating successful healing.

## Conclusions

Miller’s technique of frenectomy stands out among the various clinically used methods due to its strategic approach and superior esthetic outcomes. One of the most significant advantages of this technique is the minimal scarring it produces, particularly when compared to conventional frenectomy methods. By repositioning the incision and healing process away from the midline, Miller’s technique effectively shifts the scar from the diastema area to a less visible site. This not only enhances the esthetic result but also eliminates one of the major impediments to successful diastema closure, the formation of fibrotic scar tissue in the midline. Furthermore, the method promotes better tissue healing, maintains optimal vestibular depth, and facilitates easier orthodontic or restorative closure of the diastema without interference from residual frenum tension or unsightly scar formation. Overall, Miller’s frenectomy offers both functional and cosmetic benefits, making it a preferred technique in modern periodontal and orthodontic practice.
